# Exercise Training Attenuates Upregulation of p47^phox^ and p67^phox^ in Hearts of Diabetic Rats

**DOI:** 10.1155/2016/5868913

**Published:** 2016-02-16

**Authors:** Neeru M. Sharma, Brandon Rabeler, Hong Zheng, Eugenia Raichlin, Kaushik P. Patel

**Affiliations:** ^1^Department of Cellular & Integrative Physiology, University of Nebraska Medical Center, Omaha, NE 68198, USA; ^2^Division of Cardiology, University of Nebraska Medical Center, Omaha, NE 68198, USA

## Abstract

Exercise training (ExT) is currently being used as a nonpharmacological strategy to improve cardiac function in diabetic patients. However, the molecular mechanism(s) underlying its beneficial effects remains poorly understood. Oxidative stress is known to play a key role in the pathogenesis of diabetic cardiomyopathy and one of the enzyme systems that produce reactive oxygen species is NADH/NADPH oxidase. The goal of this study was to investigate the effect of streptozotocin- (STZ-) induced diabetes on expression of p47^phox^ and p67^phox^, key regulatory subunits of NADPH oxidase, in cardiac tissues and determine whether ExT can attenuate these changes. Four weeks after STZ treatment, expression of p47^phox^ and p67^phox^ increased 2.3-fold and 1.6-fold, respectively, in left ventricles of diabetic rats and these increases were attenuated with three weeks of ExT, initiated 1 week after onset of diabetes. In atrial tissues, there was increased expression of p47^phox^ (74%), which was decreased by ExT in diabetic rats. Furthermore, increased collagen III levels in diabetic hearts (52%) were significantly reduced by ExT. Taken together, ExT attenuates the increased expression of p47^phox^ and p67^phox^ in the hearts of diabetic rats which could be an underlying mechanism for improving intracardiac matrix and thus cardiac function and prevent cardiac remodeling in diabetic cardiomyopathy.

## 1. Introduction

Diabetes mellitus (DM) is leading metabolic disease with the highest morbidity and mortality and according to 2014 estimate of International Diabetes Federation the disease now affects more than 387 million people worldwide. DM is associated with an increased risk of cardiovascular complications, including hypertension and coronary artery disease [1]. There is growing recognition of diabetic cardiomyopathy, a primary myocardial disease, defined as either systolic or diastolic left ventricular dysfunction in otherwise healthy diabetic person. This is one of the most serious complications of diabetes; however, the underlying mechanism/s for this dysfunction are not clearly understood [2, 3]. Originally, Rubler et al. in 1972 [4], based on postmortem findings, proposed that diabetic cardiomyopathy is secondary to underlying hyperglycemia resulting in a multitude of adverse downstream effects, including impaired myocyte calcium handling, renin-angiotensin-aldosterone activation, microangiopathy, myocardial fibrosis, and increased oxidative stress [5–7]. Mechanistically, chronic hyperglycemia exerts oxidative stress to cardiomyocytes by the production of reactive oxygen species (ROS) culminating in these pathological abnormalities [8, 9]. Indeed, a number of studies have reported increased ROS formation in cultured cells exposed to high glucose concentrations. Similarly, in animal models of diabetes, ROS are generated as natural byproducts of oxygen metabolism and their moderate levels are thought to function as intracellular signaling molecules; however, high levels of ROS are detrimental to cardiomyocytes and lead to cell death, mitochondrial dysfunction due to mitochondrial fragmentation [10], or impaired insulin signaling [11]. ROS contribute to inducing various cardiovascular complications including cardiac dysfunction accelerated by inflammation, apoptosis, and fibrosis [12–14]. Furthermore, O_2_^−^ has been shown to impair nitric oxide (NO) dependent vasodilation [15, 16] and thus enhance endothelium-dependent vasoconstriction [17, 18] and precipitate cardiomyocyte hypertrophy [19].

ROS is generated by all cell types within the heart, including cardiomyocytes, endothelial cells, vascular smooth muscle cells (VSMCs), fibroblasts, and infiltrating inflammatory cells [20]. Nicotinamide adenine dinucleotide phosphate (NADPH) oxidases (NOXs) are membrane-associated enzymes which are the primary physiological producers of O_2_^−^ [21] and ROS [20, 22]. NOXs consist of 4 major subunits: a plasma membrane-spanning cytochrome b_558_ composed of a large gp91^phox^ subunit and a smaller p22^phox^ subunit, whereas 2 cytosolic subunits are p47^phox^ and p67^phox^. Endothelial cells (ECs) and myocytes express all four components of NADPH oxidase [23, 24], while VSMCs express all of the subunits with the exception of p67^phox^ subunit [25]. The low molecular weight G protein rac2 (in some cells rac1) participates in the assembly of the active complex, and a second G protein, rap1A, is thought to be involved in the deactivation of NADPH oxidase enzyme activity.

DM is associated with increased levels of Ang II [26–30] and numerous studies have shown that Ang II upsurges production of O_2_^−^ by activating NADPH oxidases [31–34] in VSMCs [35–37], adventitial fibroblasts [32, 38], ECs [33, 39], and cardiomyocytes [34]. Additionally, hyperglycemia, a key clinical manifestation of diabetes, also produces ROS via NADPH oxidase activation by glycation end products [40], while blocking of ROS formation is known to prevent hyperglycemic damage [41]. In rat ventricular myocytes, high glucose conditions induced cardiac contractile dysfunction and cardiomyopathy occurs via Ang II type 1 receptor (AT_1_R) mediated activation of NADPH oxidase [27, 28, 30]. High glucose conditions may also increase the activity of NADPH oxidase by increasing the expression of its various subunits resulting in increased production of ROS [8, 42]. Infusion of Ang II has also been shown to increase NADPH oxidase activity and O_2_^−^ production by increasing expression of p67^phox^ and gp91^phox^ in aortic adventitial cells [32]. Exposure of ventricular myocytes to high glucose also increases expression of p47^phox^ and production of ROS and these increases were blocked by AT_1_R antagonist, L-158,809 [42]. Additionally, Hink et al. [43] found increased NADPH oxidase activity and a 7-fold increase in gp91^phox^ mRNA in aortic tissue from rats with streptozotocin- (STZ-) induced diabetes.

Aerobic exercise of moderate intensity and frequency is one of a number of cardiac rehabilitation programs prescribed to patients with chronic heart failure [44]. Exercise training (ExT) reduces blood glucose, body fat, and insulin resistance and improves glycemic control, lipid metabolism, and baroreflex sensitivity in diabetes [45–48]. Additionally, ExT lowers plasma Ang II levels [49–51] and reduces renal sympathetic nerve activity and arterial pressure responses to Ang II [50–53]. Previous studies have also shown that ExT increases expression of superoxide dismutase (SOD), catalase, and glutathione in cardiac tissues [49, 54–56]. The ability of these antioxidant enzymes to reduce ROS has also been well documented [57, 58]. However, the impact of ExT on NADPH oxidase activity and expression of its subunits in diabetes is not entirely clear. These findings have led us to investigate whether diabetes-induced cardiac dysfunction may be due in part to increased expression of p47^phox^ and p67^phox^ and further whether ExT could improve/attenuate the increased expression of these subunits induced by diabetes.

## 2. Materials and Methods

### 2.1. Induction and Verification of Experimental Diabetes

Animals used for this study were approved by the Animal Care and Use Committee of the University of Nebraska Medical Center and all experiments were conducted according to the APS's* Guiding Principles for Research Involving Animals and Human Beings* and the* National Institutes of Health Guide for the Care and Use of Laboratory Animals*. Male Sprague-Dawley rats weighing between 180 and 190 g were purchased from Sasco Breeding Laboratories (Omaha, NE). After 1 week of acclimatization in housing facilities diabetes was induced by a single intraperitoneal injection (65 mg/kg) of streptozotocin (STZ, Sigma) in a 2% solution of cold 0.1 M citrate buffer (pH 4.5). Onset of diabetes was identified by polydipsia, polyuria, and blood glucose levels >250 mg/dL. Control animals were injected with citrate buffer containing no STZ. Throughout the study, animals were housed in pairs (similar weights to minimize dominance) at 22°C with fixed 12 h light/dark cycles and humidity at 30–40%. Laboratory chow (Harlan, Madison, WI) and tap water were available* ad libitum*. Experiments were performed 4 weeks after the injection of STZ or vehicle.

### 2.2. Exercise Protocol

After one week, rats in each of control and diabetic groups were randomly divided into two groups. One subgroup of control and one subgroup of diabetic rats remained sedentary, while the other two subgroups were subjected to ExT according to the protocols used by Musch and Terrell [59] with modifications [60] for three weeks after one week after diabetes induction. The resulting four subgroups of animals were referred to as nonexercised controls (Sed-control, *n* = 16), diabetic nonexercise (Sed-Dia, *n* = 16), exercise trained controls (ExT-control, *n* = 20), and exercise trained diabetic (ExT-Dia, *n* = 20). During the training period, rats were exercised between 5 and 15 min/day at an initial treadmill speed of 10 m/min up a 0% grade for 5 days. In order to ensure a significant endurance ExT regimen, the treadmill grade and speed were gradually increased to 10% and 25 m/min, respectively, and the exercise duration was increased to 60 min/day. Animals demonstrating the ability to run steadily on the treadmill with very little or no prompting (with electrical stimulation) were used in the study. The Sed-control and Sed-Dia rats were treated similarly to the ExT-control and ExT-Dia subgroup and handled daily except for the treadmill running.

### 2.3. Sample Collection

At the end of the* in vivo* protocol, animals in all four subgroups (Sed-control, Sed-Dia, ExT-control, and ExT-Dia) were anesthetized using overdose of pentabarbital (65 mg/kg, i.p.). Chest cavities were opened and hearts were removed, quick-frozen by dropping into liquid nitrogen, and stored at −80°C. Soleus muscle from hind legs was also removed, quick-frozen, and stored at −80°C.

### 2.4. Citrate Synthase Assay

The efficacy of ExT was assessed by measuring citrate synthase activity in whole muscle homogenate as previously described [51, 61]. Citrate synthase activities were normalized to total protein content and reported as micromoles per milligram protein per minute.

### 2.5. Semiquantitative RT-PCR

Relative levels of p47^phox^ and p67^phox^ in left and right ventricles and atria tissues were determined using semiquantitative RT-PCR. One hundred micron (100 *μ*m) thick coronal sections were cut on a cryostat from the left and right ventricles and a 15-gauge needle stub was used to punch atrial samples. Total RNA from left ventricle, right ventricle, and atrial tissue was isolated by TRI Reagent (MRC) method as per the manufacturer's instructions as previously described [62, 63]. Equivalent amounts of total RNA (1 *μ*g) from each of Sed-control, Sed-Dia, ExT-control, and ExT-Dia rats were then reverse-transcribed for 40 min at 37°C in the presence of 1.5 *μ*M of random hexamers and 100 U of MMLV-Reverse transcriptase. Primers for p47^phox^ and p67^phox^ (200 nM) were used in polymerase chain reactions to determine the amount of transcripts in each sample. *β*-actin was coamplified as an internal control. After 10 min of denaturing at 94°C, the amplification was performed at 94°C for 1 min, at 56°C for 1 min, and at 72°C for 1 min for 33 cycles with the final extension at 72°C for 10 min. At the end of the reaction, 7 *μ*L from each PCR reaction was mixed with Blue/Orange loading dye (Promega) and electrophoresed for 45 min at 100 V using 1% agarose gels containing ethidium bromide. The gels were visualized with gel doc system (Kodak ID gel Imager). Band intensities were then analyzed with the Kodak analysis software and normalized to that of their respective *β*-actin bands. Oligo-primers for PCR were synthesized in-house at University of Nebraska Medical Center. Sequences of primers used were as follows: p47^phox^: 5′-ACCTGTCGGAGAAGGTGGT (forward), 5′-TAGGTCTGAAGGATGATGGG (reverse); p67^phox^: 5′-AGGACTATCTGGGCAAGGC (forward), 5′-GCTGCGACTGAGGGTGAAT (reverse); *β*-actin 5′-GGGAAATCGTGCGTGACATT (forward), 5′-CGGATGTCAACGTCACACTT (reverse).

### 2.6. Western Blotting

Western blot analyses were used to determine p47^phox^ and p67^phox^ in the left ventricle, right ventricle, and atria tissues in the four subgroups of rats. Protein was extracted from the heart after homogenization in RIPA buffer (cat. number BP-115, Boston BioProducts, Worcester, MA, USA) supplemented with 1 mM phenylmethylsulfonyl fluoride and protease inhibitor cocktail. Collagen III levels were measured as an index of myocardial stiffness (diabetic cardiomyopathy) in atrial tissue. 30–40 *μ*g of each protein sample was mixed with an equal volume of 4% SDS sample buffer, fractionated on 7.5% polyacrylamide-sodium dodecyl sulfate gel, and transferred to a PVDF membrane (Millipore). After transfer, the membranes were blocked with 5% nonfat dry milk powder in TBST (20 mM Tris/HCl, pH 7.4, 150 mM NaCl, 0.1% (v/v) Tween 20) at room temperature for 1 h. Subsequently, membranes were probed with a primary anti-p47^phox^ rabbit polyclonal or anti-p67^phox^ rabbit polyclonal or anti-collagen III goat polyclonal antibody from Santa Cruz Biotechnology overnight at 4°C followed by incubation with a corresponding peroxidase-conjugated secondary antibody for one hour. The signals were visualized using an enhanced chemiluminescence (Pierce Chemical, Rockford) and detected by a UVP digital imaging system. ImageJ-NIH program was used to quantify the signal.

### 2.7. Statistical Analysis

Data was presented as mean ± SEM and subjected to Student-Newman-Keuls test. *P* values < 0.05 were considered to indicate statistical significance.

## 3. Results

### 3.1. General Characteristics of Animals

Table 1 summarizes the characteristics of the Sed-control, Sed-Dia, ExT-control, and ExT-Dia animals used in the present study. Mean body weight of all animals at the start of the study was 186 ± 2 g. As shown in Table 1, after 28 days, the mean body weight of Sed-control animals increased to 323 ± 7 g, whereas the mean body weight of ExT-control animals was 226 ± 2 g. Mean body weight of sedentary and exercised trained diabetic animals increased modestly to 209 ± 7 and 208 ± 12 g, respectively. In the animals injected with citrate buffer only, mean blood glucose levels did not change significantly during the study (72.0 ± 6.0 mg/dL at start and 78.0 ± 7.0 mg/dL at the time of sacrifice). Sed-Dia animals had significant high blood glucose levels at the time of sacrifice, 362 ± 18 mg/dL, whereas ExT had significantly lowered blood glucose levels, 315 ± 11 mg/dL. We have used citrate synthase activity as a marker of increased aerobic metabolism in the soleus muscle. The enzyme citrate synthase catalyzes the formation of citrate from acetic acid and oxaloacetic acid in the first step of Krebs cycle. Regular exercise increases the size and number of skeletal muscle mitochondria to increase respiratory capacity of the muscle. Increased mitochondria translate to increased citrate synthase activity, reflecting the increased activity of muscle associated with ExT [51, 61]. Cardiac muscle does not increase respiratory capacity in response to ExT as skeletal muscle does [64]. Therefore measuring the citrate synthase activity in soleus muscle is extensively used as a metabolic marker in assessing oxidative and respiratory capacity. In the current study, Sed-control and diabetic animals had a lower citrate synthase activity (4.58 ± 0.34 *μ*mol/g/min and 4.01 ± 0.42 *μ*mol/g/min, resp.) than ExT animals (6.93 ± 0.56 *μ*mol/g/min for ExT-control and 6.94 ± 0.56 *μ*mol/g/min for ExT-diabetic). These data also indicate that similar level of ExT was performed in the two groups of animals.

### 3.2. Increased Expression of p47^phox^ and p67^phox^ in Left Ventricle Alleviated with ExT in Diabetic Animals

**Figure 1:**
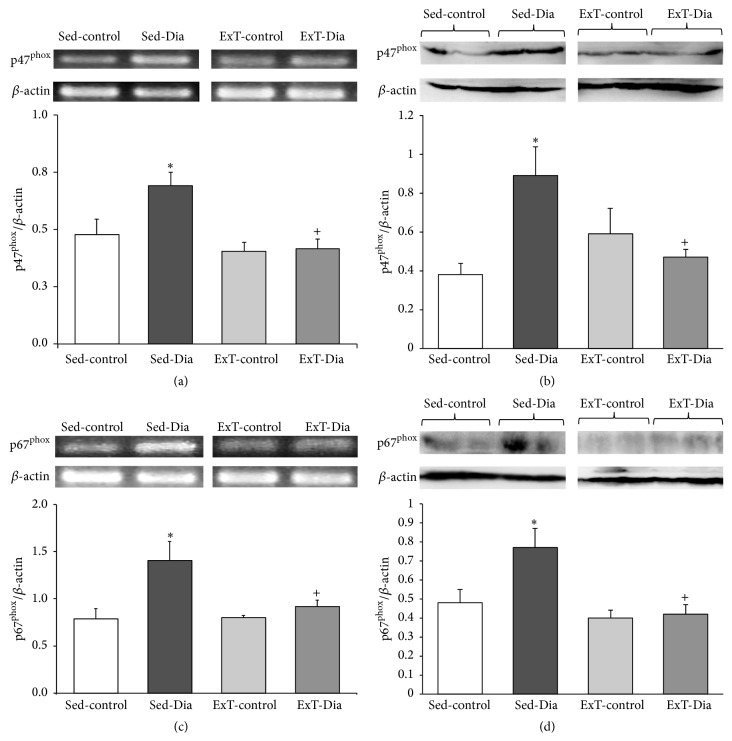
Expression of left ventricle p47^phox^ and p67^phox^ in Sed-control, Sed-Dia, ExT-control, and ExT-Dia animals. (a) RT-PCR of p47^phox^: top: a representative gel; bottom: quantification of p47^phox^ transcript normalized to *β*-actin as loading control. *n* = 10 in each group. (b) Western blot analysis of left ventricle p47^phox^ in four groups: top: a representative gel; bottom: bar graph of summary data of densitometry analyses of p47^phox^ protein level normalized to actin for loading variations. *n* = 6–8 in each group. Values represent mean ± S.E. (c) RT-PCR of left ventricle p67^phox^ in Sed-control, Sed-Dia, ExT-control, and ExT-Dia animals: top: a representative gel; bottom: quantification of p67^phox^ transcript normalized to *β*-actin as loading control. *n* = 6 in each group. (d) Protein expression of p67^phox^ in four groups: top: a representative Western blot; bottom: densitometry analysis of p67^phox^ protein normalized to *β*-actin as loading control. *n* = 3-4 in each group. Values are represented as mean ± S.E. ^*∗*^ *P* < 0.05 versus Sed-control. ^+^ *P* < 0.05 versus Sed-Dia group.

Expressions of p47^phox^ and p67^phox^ were determined by semiquantitative RT-PCR and Western blot using actin as an internal reference in four groups of rats, namely, Sed-control, Sed-Dia, ExT-control, and ExT-Dia (Figure 1). There were significantly increased transcripts of p47^phox^ I (45.8% increase) and protein (2.3-fold) in the left ventricle of 4-week Sed-Dia versus 4-week Sed-control rats (Figures 1(a) and 1(b)). Sed-Dia animals also demonstrated increased steady-state levels of mRNA encoding p67^phox^ (increased 78.1% of the 4-week Sed-control) (Figure 1(c)) and protein (1.6-fold) (Figure 1(d)) in the left ventricle. Three weeks of ExT, initiated after 1 week of diabetes, reduced mRNA and protein levels of p47^phox^ and p67^phox^ in the left ventricle of the heart to levels similar to those of the ExT controls (≈40–50% less than Sed-Dia).

### 3.3. Increased Expression of p47^phox^ and p67^phox^ in Right Ventricle Alleviated with ExT in Diabetic Animals

**Figure 2:**
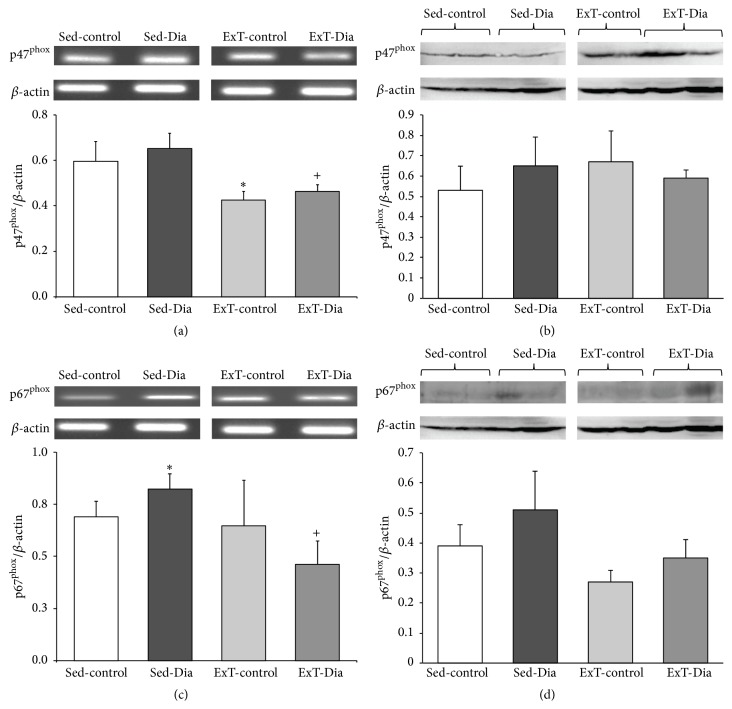
Expression of right ventricle p47^phox^ and p67^phox^ in Sed-control, Sed-Dia, ExT-control, and ExT-Dia animals. (a) RT-PCR of p47^phox^: top: a representative gel; bottom: bar graph shows quantification of densitometry analysis of p47^phox^ normalized to *β*-actin. *n* = 10 in each group. Values represent mean ± S.E. (b) Protein expression of p47^phox^ in four groups: top: a representative gel; bottom: densitometry analyses of p47^phox^ protein level normalized to actin. *n* = 3-4 in each group. Values represent mean ± S.E. (c) RT-PCR of right ventricle p67^phox^ in Sed-control, Sed-Dia, ExT-control, and ExT-Dia animals: top: a representative gel; bottom: quantification of p67^phox^ transcript normalized to *β*-actin as loading control. *n* = 6 in each group. (d) Protein expression of p67^phox^ in four groups: top: a representative Western blot; bottom: densitometry analysis of p67^phox^ protein normalized to *β*-actin as loading control. *n* = 3-4 in each group. Values are represented as mean ± S.E. ^*∗*^ *P* < 0.05 versus Sed-control. ^+^ *P* < 0.05 versus Sed-Dia group.

Similar to the left ventricle, 4 weeks of diabetes also increased p47^phox^ mRNA and protein expression in the right ventricle, albeit not reaching statistical significance compared to Sed-control (Figure 2(a)). Steady-state levels of mRNA encoding p67^phox^ in the right ventricle of the heart increased slightly (17.7%). However, there were no significant differences in p67^phox^ protein expression between the two groups (Figures 2(c) and 2(d)). Three weeks of ExT regimen attenuated the increase in steady-state levels of mRNA encoding p47^phox^ to similar levels in ExT-control and ExT-Dia groups.

### 3.4. Increased Expression of p47^phox^ and p67^phox^ in the Atria Alleviated with ExT in Diabetic Animals

**Figure 3:**
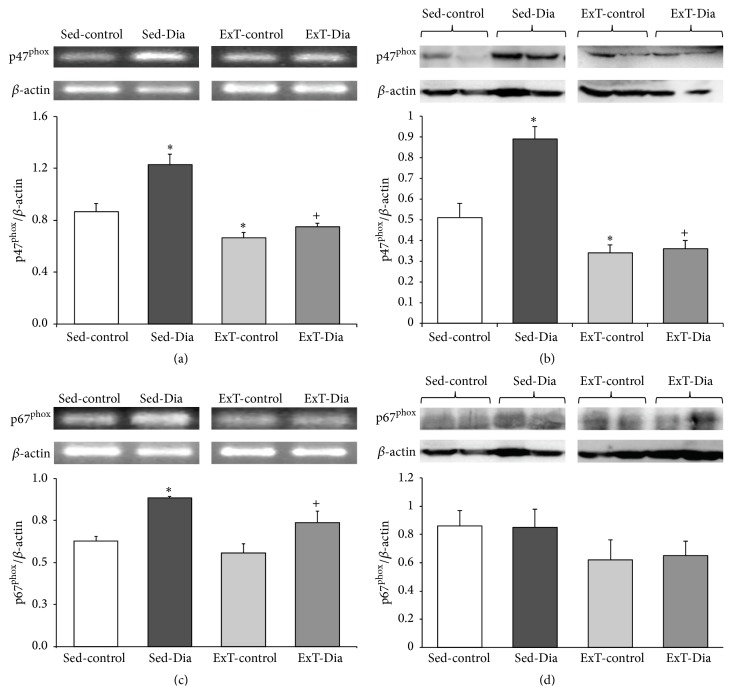
Expression of atrium p47^phox^ and p67^phox^ in Sed-control, Sed-Dia, ExT-control, and ExT-Dia animals. (a) RT-PCR of p47^phox^: top: a representative gel; bottom: bar graph shows quantification of densitometry analysis of p47^phox^ normalized to *β*-actin. *n* = 10 in each group. Values represent mean ± S.E. (b) Protein expression of p47^phox^ in four groups: top: a representative gel; bottom: densitometry analyses of p47^phox^ protein level normalized to actin. *n* = 6–8 in each group. Values represent mean ± S.E. (c) RT-PCR of atrium p67^phox^ in Sed-control, Sed-Dia, ExT-control, and ExT-Dia animals: top: a representative gel; bottom: quantification of p67^phox^ transcript normalized to *β*-actin as loading control. *n* = 6 in each group. (d) Protein expression of p67^phox^ in four groups: top: a representative Western blot; bottom: densitometry analysis of p67^phox^ protein normalized to *β*-actin as loading control. *n* = 3-4 in each group. Values are represented as mean ± S.E. ^*∗*^ *P* < 0.05 versus Sed-control. ^+^ *P* < 0.05 versus Sed-Dia group.

As shown in Figure 3, steady-state levels of mRNA encoding p47^phox^ and p67^phox^ were increased significantly in atria of the diabetic group. The increase was 30.1% for p47^phox^ and 35.5% for p67^phox^ over sedentary controls (Figures 3(a) and 3(c)). As expected, three weeks of ExT, initiated after 1 week of diabetes, significantly lowered mRNA levels of p47^phox^ and p67^phox^ in the atria of ExT-Dia heart compared to Sed-Dia group. Protein expression of p47^phox^ was increased 74%, while p67^phox^ protein expression increased but did not reach statistical significance (Figures 3(b) and 3(d)). Interestingly, p47^phox^ mRNA and protein expression in ExT-control and ExT-Dia atria was decreased to almost similar levels suggesting that ExT attenuated the increase in steady-state levels of p47^phox^ expression in atria.

### 3.5. Increased Expression of Arterial Collagen III Is Alleviated with ExT in Diabetic Animals

**Figure 4:**
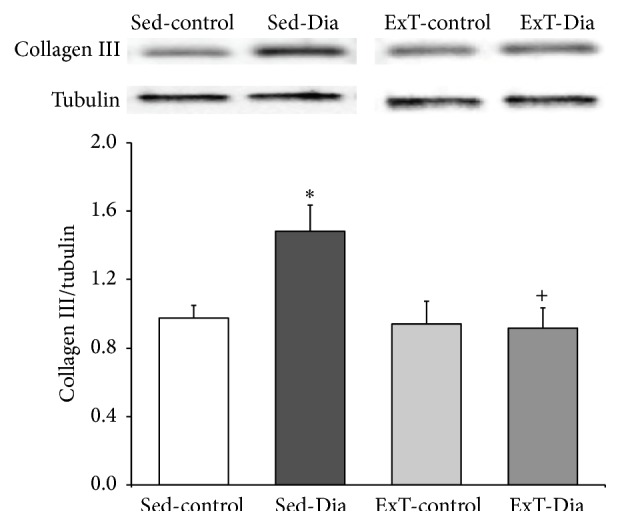
Collagen III expression in atria: top: representative Western blot showing collagen III protein expression in Sed-control, Sed-Dia, ExT-control, and ExT-Dia animals; bottom: densitometry analysis of collagen III expression normalized to *β*-tubulin as loading control. *n* = 6 in each group. Values represent mean ± S.E. ^*∗*^ *P* < 0.01 versus Sed-control group. ^+^ *P* < 0.05 versus Sed-Dia group.

As shown in Figure 4, expression of the extracellular matrix protein collagen III significantly increased by 53% in 4 weeks Sed-Dia group. This increase in steady-state levels of collagen III suggests increasing ventricular stiffening, a characteristic feature of diabetic cardiomyopathy. Three weeks of ExT initiated after 1 week of STZ-induced diabetes attenuated the increase in collagen III protein expression (38%) in the diabetic group at the same level as control, while three weeks of ExT had no effect on collagen III expression in nondiabetic control animals.

## 4. Discussion

The principal finding of the present study is that expression of the cytosolic subunits of NADPH oxidase, p47^phox^ and p67^phox^, is upregulated in hearts of STZ-induced diabetic rats and the increased expression was attenuated with ExT. Since ExT was initiated after 1 week of diabetes, it is likely that it is preventing or minimizing rather than reversing the increase in expression induced by diabetes. Previously, we have reported that Sed-Dia animals demonstrated significant reductions in fractional shortening, ejection fraction, stroke volume, and cardiac output compared with Sed-control animals and ExT (for 3 weeks) implemented 4 weeks after the onset of diabetes significantly increased percent ejection fraction, attenuated the increase in left ventricular end-systolic diameter, and improved dP/dt and isoproterenol induced increase in dP/dt in the diabetic group [60]. It is possible that these changes in expression of NADPH oxidase subunits in the heart are associated with improvement in cardiac function in diabetes.

There is increasing information that mitochondria and NADPH oxidase play a fundamental role in ROS production in the diabetic heart [12]. Furthermore, ROS-mediated increase in peroxynitrite formation induces apoptosis in cardiomyocytes* in vitro* and in the myocardium* in vivo* as shown by Levrand et al. [65] suggesting that oxidative stress is a common mediator for apoptosis induced cardiac damage in diabetic rats [13]. Our data suggest that the increased oxidative stress levels observed in diabetic hearts may be in part due to the upregulation of NADPH oxidase subunits and this change can be alleviated by ExT. These results suggest that beneficial effects of ExT may involve improvement in oxidative stress commonly observed in diabetic cardiomyopathy.

Diabetic cardiomyopathy, one of the leading causes of increased morbidity and mortality in the diabetic population, is characterized by the prolongation of action potential duration, delayed cytosolic Ca^2+^ clearance, and impaired systolic and diastolic left ventricular function [42, 60]. Several factors are believed to contribute to the pathogenesis of diabetic cardiomyopathy, including insulin resistance, enhanced renin-angiotensin system activation, hyperglycemia, and damage from ROS [66]. These pathophysiological factors associated with diabetes, including hyperglycemia and elevated Ang II levels, have been shown to increase the expression of NADPH oxidase subunits and the enzyme's activity [32, 43, 67] leading to myocardial oxidative stress. A pivotal role of NADPH oxidase in Ang II-induced cardiac hypertrophy and interstitial fibrosis was demonstrated by using mice with targeted disruption of the NADPH oxidase subunit gp91^phox^ [34]. p47^phox^ subunit of NADPH oxidase has also been demonstrated to play a key role in the enzyme's activation by agonists. Using p47^phox^ knockout mice and p47^phox^ cDNA transfection and antisense techniques, Li et al. [68] demonstrated the necessity of p47^phox^ subunit for the activation of NADPH oxidase when exposed to the agonists Ang II, TNF-*α*, or phorbol 12-myristate 13-acetate, an activator of protein kinase C. The phosphorylation of p47^phox^ is believed to induce conformational changes that facilitate its binding to the other cytosolic subunits, including p67^phox^, and their subsequent binding to the membrane-bound cytochrome b_558_ to allow optimal O_2_^−^ production [69]. Precise mechanisms underlying agonist-induced nonphagocytic NADPH oxidase activation are not yet understood, but it is clear that both acute protein modification and chronic changes in expression levels may be involved.

Our findings indicate that the cardiac tissue expressions for p47^phox^ and p67^phox^ are upregulated in the diabetic rats. This upregulation was greatest in the left ventricle and atria, while it was slightly elevated in the right ventricle of the diabetic group. These findings are consistent with clinical data showing an association of DM with an increased risk of systolic, diastolic, and any left ventricular dysfunction and the relative sparing of the right ventricle [1, 2]. In ventricular myocytes, it was reported that hyperglycemic conditions increase the production of ROS via an enhanced expression of p47^phox^ protein, which could be blocked by an AT_1_R antagonist [42]. Cifuentes et al. [32] have also shown that Ang II infusion, via the AT_1_R activation, induces an enhanced expression of p67^phox^ and gp91^phox^ proteins in aortic adventitia. Additionally, an increased NADPH oxidase activity and a 7-fold upregulation of gp91^phox^ mRNA in aortic tissue have been reported in STZ-induced diabetic rats [43]. Furthermore, increased NADPH oxidase activity and collagen 3 were demonstrated in pathogenesis of the heart failure as seen in hearts of Duchenne muscular dystrophy rat model [70] and also a significant increase in collagen III was found in endomyocardial biopsies obtained from patients with DM [71]. Ang II has also been shown to stimulate collagen I and collagen III in cardiac fibroblast, and apocynin, an inhibitor of NADPH oxidase, prevents this initiation suggesting a critical role for ROS in cardiac remodelling [72]. Although a medical treatment with different antioxidant has been proposed for diabetic cardiomyopathy for decades, unfortunately, such treatments have failed to attenuate cardiac dysfunction or improve outcomes [73]. This may be due to lack of improving the ROS load in an appropriate measure in the myocardium rather than an overall reduction in ROS.

Physical activity is widely accepted as a key element in the prevention of type 2 diabetes [74] and also has beneficial effects in patients with established heart disease [75], although the physiological mechanisms explaining how physical activity promotes health remain to be fully elucidated. ExT maintains cardiac output by blunting diabetes-induced bradycardia and the reduction in force of myocardial contractility [60]. ExT has been shown to elicit a number of beneficial physiological changes in animals and clinical studies. These include (1) a reduction in blood glucose, body fat, and insulin resistance [45, 47], (2) improved glycemic control and lipid metabolism [45, 76], (3) improved baroreflex sensitivity [46], (4) reduced plasma Ang II levels [49–51, 54], and (5) reduced renal sympathetic nerve activity and arterial pressure responses to Ang II [50, 52, 54, 77]. Additionally, the ability of ExT to upregulate antioxidant enzyme expression and activity in the cardiovascular system has been clearly demonstrated [48, 54, 55, 78, 79]. Previously, our lab showed ExT initiated after the onset of diabetes blunts primarily beta(1)-adrenoceptor expression loss and improves cardiac function in diabetic rats [60].

The current study adds to this literature by demonstrating for the first time that ExT significantly reduces p47^phox^ mRNA expression in the left and right ventricles and atria of diabetic rats. In diabetic rats, ExT significantly reduced p67^phox^ mRNA expression of the left ventricle and attenuated the increased p67^phox^ message in the atria. The increase in NADPH oxidase subunit expression observed in the diabetic condition and its attenuation by ExT may occur via a variety of mechanisms. Hyperglycemia, enhanced renin-angiotensin system activity, and elevated levels of circulating cytokines are all clinical manifestations associated with the diabetic condition, and each is known to promote NADPH-derived O_2_^−^ production. TNF-*α*, a cytokine that activates transcription factors which induce the expression of genes involved in inflammation and cell growth, has been identified as an agonist for NADPH oxidase [68]. Levels of TNF-*α* are increased in response to both hyperglycemic conditions and elevated Ang II levels, and the increase is mediated via AT_1_R [68, 80]. Hyperglycemia and elevated Ang II levels have been clearly shown to upregulate the expression of NADPH oxidase subunits and increase NADPH oxidase activity and O_2_^−^ production [32, 39, 67, 81]. Based on these findings, it appears that hyperglycemia and elevated Ang II associated with diabetes work via the AT_1_ receptor to increase TNF-*α* levels and NADPH oxidase activity, while ExT suppresses expression of TNF-alpha and thus offers a potential protection against TNF-alpha-induced insulin resistance [82] and increased ROS production [83, 84].

Our study provides evidence that increase in oxidative stress, specifically in left ventricle, may be due to an enhanced transcription as well as translation of p47^phox^ and p67^phox^ subunits of NADPH oxidase. Expression of p67^phox^ was increased at transcription level but not at the protein level in atria of diabetic rats suggesting that increased transcription did not contribute to changes in protein expression in this tissue. Translocation of cytosolic subunits (p47^phox^, p67^phox^, p40^phox^, and rac1) and their association with membrane-bound cytochrome b_558_ (consisting of one p22^phox^ and one gp91^phox^ subunit) follows during acute oxidase activation [85]. In present studies perhaps there was only translocation of p67^phox^ in the atria of diabetic heart rather than overall expression of synthesis, a possibility that remains to be explored.

The numerous physiological benefits of ExT offer several mechanisms via which the expression and activity of NADPH oxidase may be attenuated in diabetes. ExT has been shown to normalize diabetes-related elevations in blood glucose [45–47], plasma Ang II [49, 50, 53], and TNF-*α* [83, 84]. Normalizing these factors could lower levels of ROS-inducing agonists, thus dampening NADPH oxidase activity. Additionally, ExT has repeatedly been shown to upregulate antioxidant expression and activity, including SOD, catalase, and glutathione, in aortic and cardiac tissues [48, 49, 54, 56, 78, 79]. However, while the literature provides some insight, the specific mechanism by which ExT lowered the mRNA expression of p47^phox^ and p67^phox^ in our current study remains to be elucidated.

## 5. Conclusions

**Figure 5:**
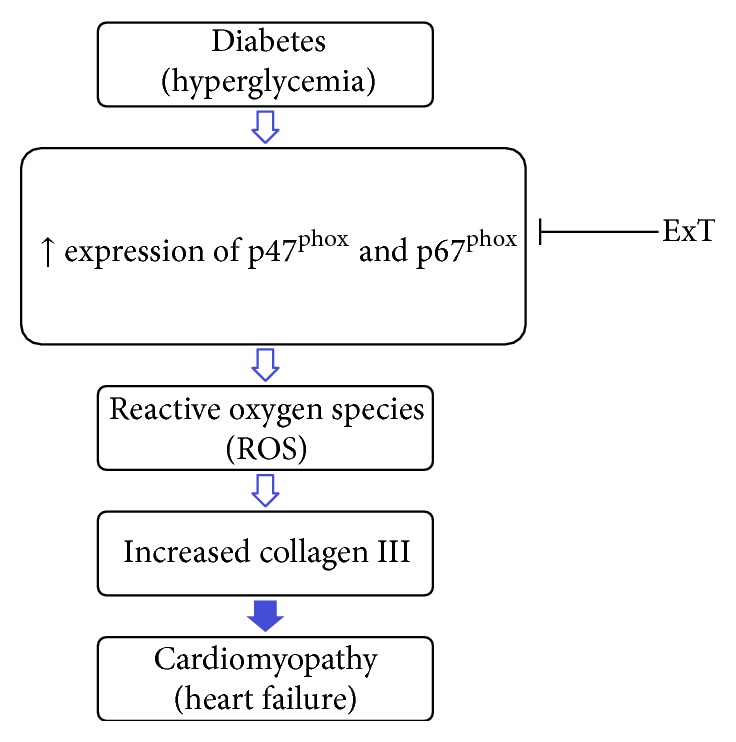
Amelioration of cardiomyopathy by exercise training. Hyperglycemia induces overexpression of cytoplasmic subunits of NADH oxidase (p47^phox^ and p67^phox^) in left ventricle. Overexpression of these subunits exhibits high reactive oxygen species and leads to increased collagen III and hence cardiomyopathy. Exercise training (ExT) mitigates the expression of p47^phox^, and p67^phox^ consequently ameliorates heart dysfunctions.

In summary, our data show that the mRNA expressions of the NADPH oxidase subunits p47^phox^ and p67^phox^ are upregulated in cardiac tissue in the diabetic condition. Furthermore, our data show that ExT attenuates the upregulated expression of these NADPH oxidase subunits and normalizes the increased collagen III levels (Figure 5) and provides support with a potential mechanistic link for exercise training as being an effective nonpharmacological tool in regulating oxidative stress levels in the diabetic heart. Future areas of research will need to focus on understanding some of the mechanisms involved in diabetic cardiomyopathy and the therapeutic strategies such as ExT to halt or slow its progression.

## Figures and Tables

**Table 1:** Characteristics of the Sed-control, Sed-Dia, ExT-control, and ExT-Dia groups. *n* = 10 in each group. Values represent mean ± S.E. ^*∗*^ *P* < 0.01 versus Sed-control. ^#^ *P* < 0.05 versus Sed-Dia group.

	Sed-control	Sed-Dia	ExT-control	ExT-Dia
	(*n* = 10)	(*n* = 10)	(*n* = 10)	(*n* = 10)
Body weight (g)	323 ± 7	209 ± 7^*∗*^	226 ± 2^*∗*^	208 ± 12^*∗*^
Blood glucose (mg/dL)	72 ± 6	362 ± 18^*∗*^	78 ± 7	315 ± 11^#^
Citrate synthase (mmol/g/min)	4.58 ± 0.34	4.01 ± 0.42	6.93 ± 0.56^*∗*^	6.94 ± 0.56^*∗*^
